# Integrated Proteogenomic Analysis Reveals Distinct Potentially Actionable Therapeutic Vulnerabilities in Triple-Negative Breast Cancer Subtypes

**DOI:** 10.3390/cancers16030516

**Published:** 2024-01-25

**Authors:** Pushpinder Kaur, Alexander Ring, Tania B. Porras, Guang Zhou, Janice Lu, Irene Kang, Julie E. Lang

**Affiliations:** 1 Department of Surgery, Keck School of Medicine, University of Southern California, Los Angeles, CA 90033, USA; 2 Norris Comprehensive Cancer Center, University of Southern California, Los Angeles, CA 90033, USA; 3 Department of Medical Oncology and Hematology, University Hospital Zürich, 8091 Zurich, Switzerland; 4 Cancer and Blood Disease Institute, Children Hospital Los Angeles, University of Southern California, Los Angeles, CA 90027, USA; 5 Division of Breast Services, Department of General Surgery, Digestive Disease and Surgery Institute, Department of Cancer Biology, Lerner Research Institute, Cleveland Clinic, Cleveland, OH 44195, USA; 6 Division of Medical Oncology, Department of Medicine, University of Southern California Norris Cancer Center, University of Southern California, Los Angeles, CA 90033, USA

**Keywords:** TNBC, proteomic, genomic, transcriptomic, breast cancer

## Abstract

**Simple Summary:**

Despite the significant progress made in precision oncology and genetic analysis, there are few effective treatment options available for triple-negative breast cancer (TNBC). Developing successful treatment approaches for TNBC requires the exploitation of therapeutic vulnerabilities using a multi-dimensional framework. In this study, we analyzed protein data from the Cancer Genome Atlas (TCGA) dataset by integrating it with DNA and RNA sequencing. By working backwards from protein to RNA to DNA, we identified copy number alterations as the key genomic drivers over mutations, and therefore copy number alterations represent important potential therapeutic targets in TNBC. Our multi-omics approach aids in the identification of potentially predictive alterations in TNBC, bringing us closer to precision medicine for this aggressive disease.

**Abstract:**

Triple-negative breast cancer (TNBC) is characterized by an aggressive clinical presentation and a paucity of clinically actionable genomic alterations. Here, we utilized the Cancer Genome Atlas (TCGA) to explore the proteogenomic landscape of TNBC subtypes to see whether genomic alterations can be inferred from proteomic data. We found only 4% of the protein level changes are explained by mutations, while 21% of the protein and 35% of the transcriptomics changes were determined by copy number alterations (CNAs). We found tighter coupling between proteome and genome in some genes that are predicted to be the targets of drug inhibitors, including CDKs, PI3K, tyrosine kinase (TKI), and mTOR. The validation of our proteogenomic workflow using mass spectrometry Clinical Proteomic Tumor Analysis Consortium (MS-CPTAC) data also demonstrated the highest correlation between protein–RNA–CNA. The integrated proteogenomic approach helps to prioritize potentially actionable targets and may enable the acceleration of personalized cancer treatment.

## 1. Introduction

TNBC is a diverse group of breast cancers lacking expression of estrogen receptor (ER), progesterone receptor (PR), or human epidermal growth factor receptor 2 (HER2), and therefore lacks biomarker-directed therapeutic options that are available and have been applied with remarkable success in other breast cancer subtypes. Finding targeted therapies for TNBC remains a crucial unmet need in clinical oncology.

First described by Lehmann et al. and corroborated by subsequent studies, six TNBC molecular subtypes have been identified, which are characterized by distinct gene expression patterns and response to therapy [1,2,3,4,5]. Subsequent refinement to four molecular subtypes confirmed differences in clinical presentation and response to therapy [6,7]. Although these genomic studies have led to the rationale for ongoing clinical trials involving androgen receptor and PARP inhibitors in biomarker-selected populations, very few options currently exist for targeted therapy in TNBC. Moreover, several studies have demonstrated that hypoxia also induces genomic alterations in TNBC patients and is responsible for treatment resistance and recurrence [8,9,10,11]. Thus, there is a need for an improved understanding of actionable molecular alterations and their correlation with functional changes predicting drug response. 

Elucidating the genomic alterations, beyond deleterious mutations, underpinning oncogenic transcriptional and translational events is essential for a comprehensive understanding of tumorigenesis and potential therapeutic vulnerabilities. The Cancer Genome Atlas (TCGA) consortium has performed large-scale reverse phase protein array (RPPA)-based proteomic analysis for the quantification of proteomes and phosphoproteomes of ~171 cancer-related proteins in breast cancer [12]. The NCI Clinical Proteomic Tumor Analysis Consortium (CPTAC) further analyzed the proteome of 77 TCGA breast tumor samples using mass spectrometry [13]. Recently, we have performed an integrative genomic approach for the identification of putative actionable alterations in clinically relevant genes in breast cancer [14]. We identified 49 potentially clinically actionable genes for which drugs are either available clinically or via clinical trials in breast cancer [15]. We analyzed the TCGA whole exome sequencing (WES) and RNA sequencing (RNA Seq) data, querying for these 49 genes mutational hotspots and stratifying our findings based on the known intrinsic subtypes of breast cancer. TNBC patients harbored DNA alterations in proliferation signaling, cell cycle regulators, stem cell, and immunoregulatory targets. High-level amplifications (copy number ≥ 2) and homozygous deletion (double copy loss) in clinically actionable genes had a clear impact on gene expression. Herein, we describe an integrated proteogenomic analysis of TCGA TNBC breast cancer samples representing the four principal mRNA-defined breast cancer intrinsic subtypes described by Lehmann et al. [6].

The objective of this study was to undertake an integrated analysis of DNA mutations/CNAs with RNA and protein expression for the identification of potentially clinically actionable markers in four molecular subtypes of TNBC. We hypothesized that employing a reverse analysis approach from protein and RNA expression to DNA mutations and copy number alterations (CNAs) could serve as a foundation for identifying key driver mutations, as well as discerning the expression patterns of proteins across subtypes of TNBC.

## 2. Material and Methods

### 2.1. Data Retrieval and Ethical Approval

The TNBC proteomics data analyzed in this study were generated by the TCGA RPPA platform, including tumors from n = 33 basal-like1 (BL1), 22 basal-like 2 (BL2), 29 luminal androgen receptor (LAR), and 29 mesenchymal (M) subtypes. The RPPA methodology used for profiling TCGA tumors has been previously described [12]. The normalized protein expression and phosphoproteins RPPA data were downloaded from the TCGA TNBC dataset from the Broad Institute GDAC Firehose (https://gdac.broadinstitute.org/ (accessed on 3 August 2018)). The clinical information for the same samples was retrieved from the TCGA data portal (https://tcga-data.nci.nih.gov/tcga/ (accessed on 3 August 2018)). Twenty-three potentially actionable targets analyzed in this study overlapped with our previously defined list of 49 actionable breast cancer targets [15]. For mutations, copy number events, and normalized gene expression data from RNA-Seq, we downloaded the processed data from cBioportal [16,17], as the data analysis was based on specific genes of interest. The TNBC tumors were encoded with TCGA sample codes. The study protocol was approved by the Cleveland Clinic Institutional Review Board (IRB) Committee (Protocol no. #21-654, Approval date: 2 June 2021).

### 2.2. Bioinformatic Analysis Pipeline for Multi-Omics Data

Protein correlation analysis and visualization was performed using ClustVis (http://biit.cs.ut.ee/clustvis/ (accessed on 8 October 2022)) [18], employing principal component analysis (PCA) and hierarchical clustering of protein and phosphoprotein data. For RNA-Seq data, Z-score thresholds were set at −2 and +2 representing the standard deviations from the mean of expression values that are diploid for the gene of interest. One-tailed hypothesis testing was applied to transform the z-score into *p*-values. Morpheus (https://software.broadinstitute.org/morpheus/ (accessed on 15 October 2022)) was used to generate heatmap and clustering analysis using Euclidean distance and average linkage. The PCA plot for normalized RNA expression for TNBC subtypes was generated using the ClustVis tool. 

For DNA mutation analysis, the R package Maftools version 2.18.0 [19] was used to identify the most frequently mutated genes, to perform a cancer driver gene analysis, and to identify the enriched oncogenic signaling pathways affected by actionable alterations in TNBC tumors. The Oncodrive-FML analysis [20] was performed to generate quantile–quantile (QQ) plots for the identification of significantly mutated actionable genes among TNBC subtypes. Significant CNAs were identified by the genomic identification of significant targets in cancer (GISTIC) [21] for each TNBC subtype. GISTIC analysis provided the copy number (expressed as a log2 ratio) and CNA thresholds were defined according to the set of discrete copy number calls, which was composed of homozygous deletion (−2), hemizygous deletion (−1), low-level gain (1), and high-level amplification (2). 

### 2.3. Validation of the Proteogenomic Workflow 

To validate our findings, we analyzed mass spectrometry CPTAC data generated from 29 basal patients. We obtained mutations, RNA expression, CNAs, and protein data using cBioportal. To assess whether the proteomic changes are associated with transcriptomic changes or mutational and CNAs events, we performed Mann–Whitney tests for 9/23 markers that showed significant association between protein–RNA–CNAs and protein–mutation level in the RPPA data.

To identify TNBC-specific genetic and pharmacological vulnerabilities, we used those markers identified in our proteogenomic analysis to further select the putative biomarkers of drug sensitivity/resistance in TNBC. We extracted the drug response vector IC50 z-scores from TNBC cell lines utilizing the Genomics of Drug Sensitivity in Cancer (GDSC) database (https://www.cancerrxgene.org/ (accessed on 20 November 2023)) containing the drug sensitivity/resistance data across 1000 human cancer cell lines.

### 2.4. Statistical Analysis 

Statistical analysis was performed using GraphPad Prism 9 (GraphPad Software, San Diego, CA, USA). Multiple hypothesis testing was conducted using the two-stage linear step-up procedure of Benjamini, Kreiger, and Yekutieli [22,23,24] and by setting the false discovery rate (FDR) (Q) to 5% to generate *q*-values. The statistical significance threshold was set at an alpha level of 0.05 for all statistical tests. Spearman’s rank correlation test was conducted on protein and phosphoprotein levels and a correlation magnitude of 0.5 or higher was considered significant for positive and negative correlated pairs. The two-way ANOVA test was computed to identify TNBC subtypes-specific associations with individual protein and phosphoprotein levels. The Mann–Whitney test was applied to evaluate the association of protein expression and RNA expression as well as their correlation with somatic mutations and CNAs. The Kruskal–Wallis test was computed to characterize the variant allele fractions (VAFs) of somatic variants and allelic heterogeneity in four TNBC cancer subtypes.

## 3. Results

### 3.1. Proteomic Phenotypes and Protein–Protein Interactions in TNBC Subtypes

We analyzed the proteomic signatures of clinically relevant proteins among four TNBC molecular subtypes (BL1, BL2, LAR, and M) described by Lehmann et al. [6]. The clinical characteristics and patients’ demographics are presented in Table 1. Most of these patients had early-stage breast cancer and none had received neoadjuvant treatment.

**Figure 1:**
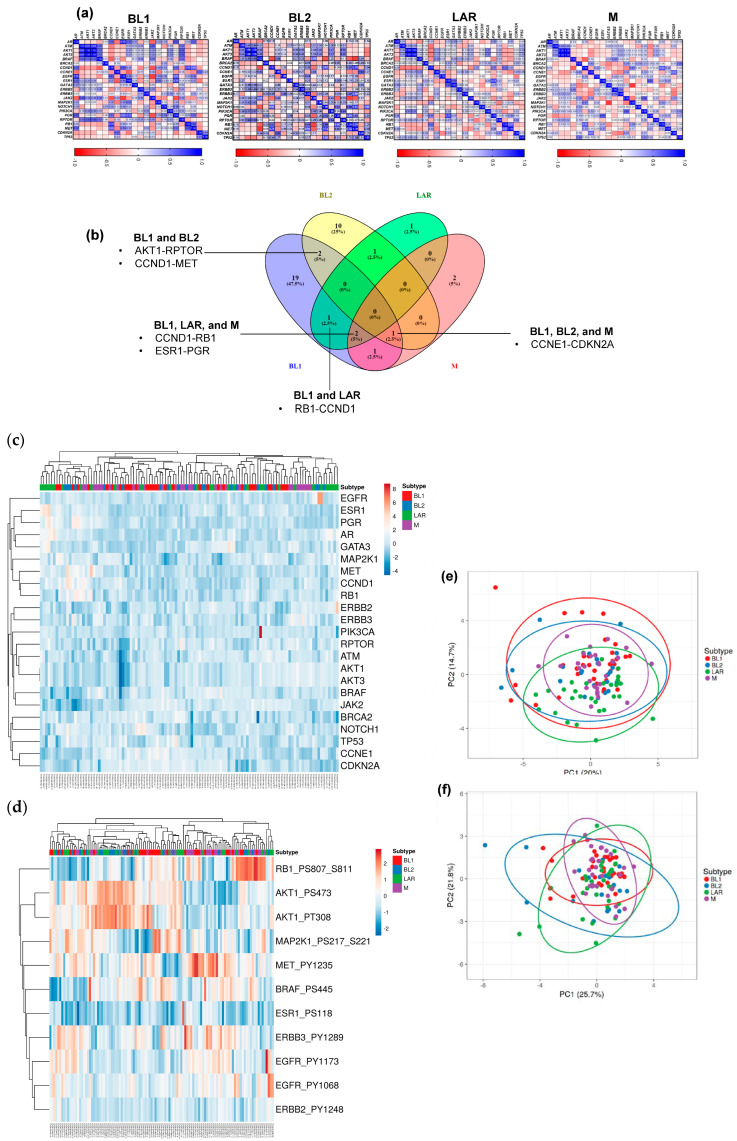
Proteomic landscape of potentially actionable proteins across TNBC subtypes. (**a**) The Spearman correlation matrix between protein–protein for each gene in BL1, BL2, LAR, and M. The blue color represents similarity in protein expression. (**b**) Venn diagram showing common gene pairs involved in protein–protein interaction in each TNBC subtype. (**c**) Heatmap showing the hierarchical clustering of TNBC subtypes for normalized expression of 23 actionable proteins. The color bar displays the protein expression values ranging from −2 (under expression) to 2 (overexpression). (**d**) Hierarchical clustering of TNBC subtypes for phosphoprotein levels of 11 actionable genes. The color bar displays the protein expression values ranging from −2 (under expression) to 2 (overexpression). (**e**) PCA plot showing no significant variation for protein expression between TNBC subtypes. Unit variance scaling is applied to rows; singular value decomposition (SVD) with imputation is used to calculate principal components. X and Y axes show PC1 and PC2 that explain 20% and 14.7% of the total variance, respectively. Prediction ellipses are such that, with a probability 0.95, a new observation from the same group will fall inside the ellipse. N = 113 data points. (**f**) PCA plot showing no significant variation for phosphoprotein levels between TNBC subtypes. Unit variance scaling is applied to rows; singular value decomposition (SVD) with imputation is used to calculate principal components. X and Y axes show PC1 and PC2 that explain 21.8% and 25.7% of the total variance, respectively. Prediction ellipses are such that, with a probability 0.95, a new observation from the same group will fall inside the ellipse. N = 113 data points.

The proteomic and phosphoproteomic landscape of 23 potentially actionable proteins and 11 phosphoproteins that are available with the TCGA RPPA data from our list of 49 actionable genes were analyzed in all four TNBC subtypes [15]. Beyond individual protein levels, protein–protein interaction analysis identified 10/23 (43%) protein pairs with statistically significant overexpression (R > 0.5, Spearman test, *p* < 0.05) and 9/23 (39%) significantly downregulated (R < −0.5, Spearman test, *p* < 0.05) protein pairs in TNBC subtypes (Figure 1a, Supplementary Table S1). Functionally, these proteins are involved in cell division and proliferation (CCND1-RB1 in BL1, LAR, and M subtypes, CCNE1-CDKN2A in BL1, BL2, and M), stem cell phenotype (RB1-MET in BL2 and LAR subtypes), and hormone receptor signaling (ESR1-PGR in BL1, LAR, and M subtypes, CCND1-MET in BL1 and LAR) (Figure 1b).

For individual protein analysis, heatmaps of unsupervised clustering analysis for all four TNBC subtypes showed similar protein and phosphoprotein expression patterns in the clustering (Figure 1c,d). None of the 23 proteins and 11 phosphoproteins were considered significant among different subtypes (Supplementary Figure S1). PCA plot of proteomics and phosphoproteomics data showed no distinct differences between different TNBC subtypes (Figure 1e,f).

Overall, our analysis demonstrated variations in the protein–protein correlation patterns across the TNBC subtype that could help in identifying effective drug combinations, but no common interacting pairs were identified in all TCGA TNBC tumors.

### 3.2. TP53 and PIK3CA Driver Genomic Alterations 

**Figure 2:**
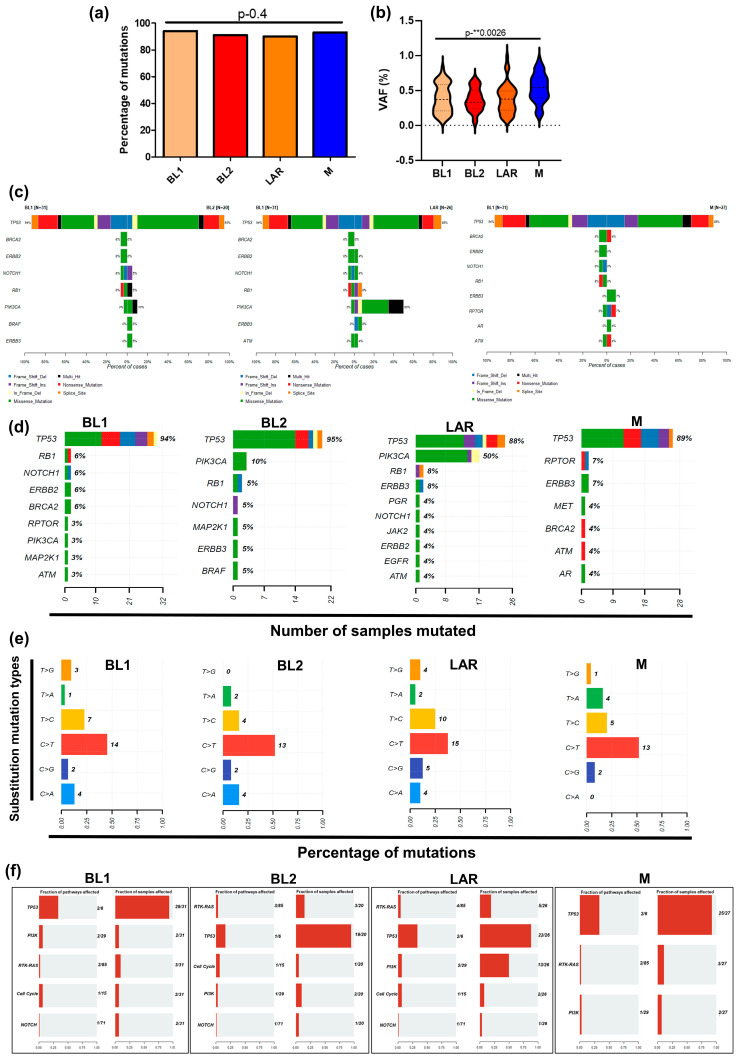
Mutational landscape of potentially actionable genes across TNBC subtypes. (**a**) Bar graph depicting the percentage of cases with mutations in actionable genes in TNBC cancer subtypes (Kruskal–Wallis test, *p* < 0.05). (**b**) Violin plot indicating substantial variation of hotspot mutations VAFs between the distinct TNBC subtypes. We applied Kruskal–Wallis test to perform an overall comparison of VAFs among each TNBC subtype (Kruskal–Wallis test, *p* < 0.05). ** means *p*-value ≤ 0.01. For post-hoc analysis, pairwise comparisons of VAFs were also performed using the Mann–Whitney test, with *p* < 0.05 considered statistically significant. (**c**) Cobar plots showing the side-by-side comparison for the most recurrently mutated genes between two TNBC subtypes (Fisher’s test on 2 × 2 contingency table). (**d**) Oncoplot generated by the R Bioconductor Maftools package displaying the 10 most frequently mutated genes, color-coded by type of mutations in breast cancer subtypes. The percentage to the right of the Oncoplot shows the percentage of samples with variants for the corresponding gene. The *x*-axis shows the total number of samples mutated and the *y*-axis shows the frequently mutated genes. By default, samples are ordered by the most frequently mutated genes. (**e**) Barplot showing the distribution of base substitutions in 4 TNBC molecular subtypes. The percentage mutations are shown on the *x*-axis and substitution mutation types are on the *y*-axis. (**f**) The “Oncogenic Pathways” module of Maftools displayed enrichment of five canonical signaling pathways showing the fraction of genes and samples affected within that pathway.

We next profiled the mutational landscape for 23 actionable proteins in all four TNBC subtypes. A total of 157 somatic variants comprising 94 missense mutations, 35 inframe mutations, 21 nonsense mutations, and 7 splice site mutations were identified, without statistically significant differences across TNBC subtypes (Kruskal–Wallis test, *p* = 0.4) (Figure 2a). However, a significant difference was observed in VAFs in hotspot mutations between the distinct subtypes (Kruskal–Wallis test, *p* = 0.003), suggesting allelic heterogeneity between TNBC subtypes (Figure 2b). 

The Oncodrive function of Maftools [25] identified TP53 as the most frequently mutated gene across all four TNBC subtypes and PIK3CA as the second most mutated gene in the LAR subtype (Figure 2c,d). No significant differences were observed in six classes of mutation types. We found that C > T transitions were the most frequent single-base substitutions in all six of the TNBC subtypes (Figure 2e). This signature was also found to be the most dominant mutation in 21 primary breast cancer genomes [26]. The OncodriveFML approach was performed for the identification of drivers by computing the functional impact (FI) score using the set of somatic mutations in a gene [20,27]. The significantly mutated genes (*q* < 0.01) were TP53 in all four TNBC subtypes, and PIK3CA in BL2 and LAR subtypes (Supplementary Table S2 and Supplementary Figure S2a). The most enriched oncogenic pathway among all subtypes was TP53, and this enrichment was found in >80% of samples. The PI3K pathways were only enriched in the LAR subtype in 50% of samples (Figure 2f). Despite the limited activity of single agents, the combinatorial therapies targeting these pathways have been evaluated in clinical trials in TNBC [28]. 

Notably, our reverse analysis approach from proteins to DNA mutations identified a significant correlation for only TP53 missense mutations with elevated protein expression, while TP53 truncating mutations were significantly associated with decreased protein levels (Supplementary Figure S2b). However, TP53 frameshift alterations did not result in decreased protein expression, but, rather, suggested increased TP53 protein expression (not significant) (Supplementary Figure S2b). No significant association was observed in samples with PIK3CA mutation and their protein expression level. 

### 3.3. Proteomic and Transcriptomic Profile of TNBC Subtypes

**Figure 3:**
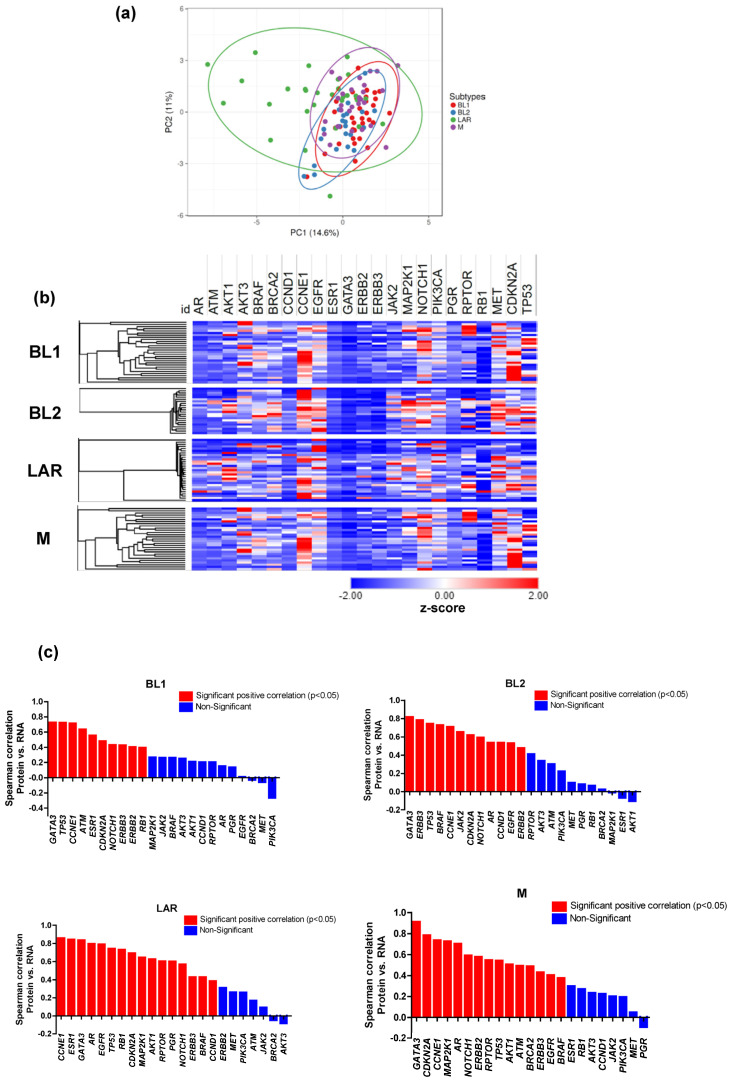
Comparison of transcriptomic change to protein change. (**a**) PCA plot of RNA-Seq data showing the variation for gene expression (z-scores) between TNBC subtypes. Unit variance scaling is applied to rows; singular value decomposition (SVD) with imputation is used to calculate principal components. X and Y axes show PC1 and PC2 which explain 14.6% and 11% of the total variance, respectively. Prediction ellipses are such that, with a probability of 0.95, a new observation from the same group will fall inside the ellipse. N = 113 data points. (**b**) Hierarchical clustering of TNBC subtypes according to the expression of 23 actionable genes. The RNA expression of the actionable genes was calculated as a z-score ranging from −2 (under expression) to 2 (overexpression). (**c**) Significant positive and negative Spearman correlation between protein–mRNA expression of 23 actionable genes across TNBC subtypes (*p* < 0.05).

We next assessed the transcriptomic features that are commonly shared or distinguish different TNBC subtypes in 23 actionable genes. The LAR subtype has a distinct gene expression pattern with higher expression levels of EGFR (*p* < 0.00001) and PIK3CA (*p* < 0.00001) (Figure 3a). Among commonly shared genes, we identified upregulated expression of proliferative signaling target (EGFR (*p* < 0.00001)) in BL1, BL2 and LAR subtypes. We also found actionable genes with RNA overexpression that distinguish TNBC subtypes. For instance, EGFR (<0.00001) and stem cell signaling targets NOTCH1 (*p* = 0.03), and MET (*p* = 0.02) characterizes BL2. The cell proliferation marker AKT3 (*p* = 0.03) characterizes BL1 subtype. Both BL1 and M subtypes also displayed increased mRNA expression levels for CDKN2A, suggesting the potential sensitivity of these two subtypes to CDK4/6 inhibitors (Figure 3b). 

To evaluate whether protein changes are explained by transcriptomic changes, we computed Spearman correlations for each actionable gene. In all TNBC subtypes, statistically significant correlations between mRNA expression and protein abundance were commonly observed in 6/23 (26%) genes (GATA3, CCNE1, CDKN2A, NOTCH1, TP53, and ERBB3) (Supplementary Table S3). The protein–mRNA concordance associated with TNBC subtypes was identified in 13/23 (56%) genes (BRAF, AR, EGFR in BL2, LAR, and M subtypes; ERBB2 in BL1, BL2, and M subtypes; ESR1 in BL1 and LAR subtypes; JAK2 in BL2; BRAC2 in M subtype; CCND1 in BL2 and LAR subtypes; ATM in BL1 and M; RB1 in BL1 and LAR; and MAP2K1, AKT1, and RPTOR in LAR and M subtypes) (Figure 3c and Supplementary Table S3). 

Overall, these results show that matched protein–mRNA and protein–protein correlations were the highest on average, followed by protein–mutation correlations. 

### 3.4. Effects of CNAs on Transcriptome and Proteome 

**Figure 4:**
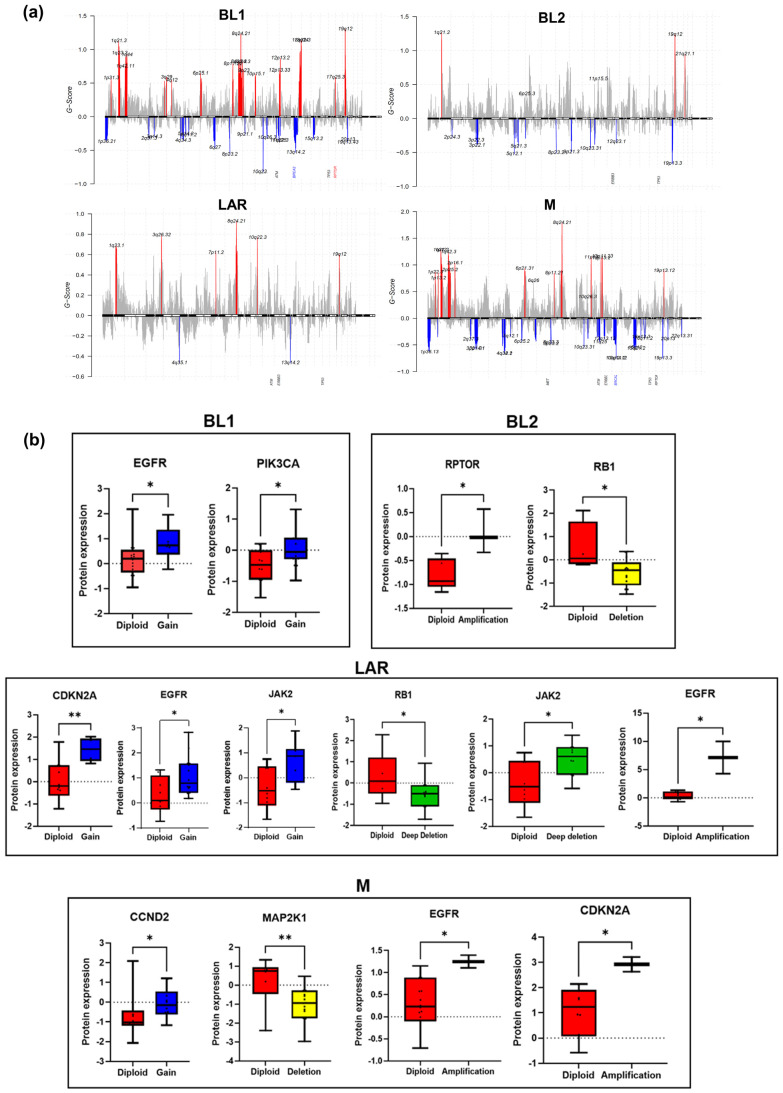
CNAs in potentially actionable genes and their effect on protein level. (**a**) GISTIC analysis of genome-wide copy number landscape showing the widespread alterations in each TNBC subtype. The *y*-axis depicts the amplification (red peaks) and deletion (blue peaks) G-score, and the *x*-axis denotes the position along the corresponding chromosome. GISTIC assigns each CNA a G-score that represents the amplification and deletion of the aberration. Significant amplified and deleted regions which contain potential actionable genes from our list are given on the *x*-axis. (**b**) Boxplots showing the protein expression level of 8 actionable genes in diploid (wild type (WT)) (red), gain (blue), amplification (black), deletion (yellow), and deep deletion (green) cases across TNBC subtypes. The *y*-axis shows protein expression level of wild type and copy number altered cases. * means *p*-value ≤ 0.05 and ** means *p*-value ≤ 0.01.

The genome-wide landscape of CNAs indicates widespread alterations across different TNBC subtypes (Figure 4a). Our analysis identified amplifications in chromosomes 1p, 1q, 8q, 10p, 11p, 12p, 13q, 19q and deletions in 2q, 3p, 5q, 6p, 8p, 10q, 11p, 11q, 12p, 12q, 13q, 19p, 20p among all TNBC subtypes. To identify the most clinically relevant CNAs in TNBC subtypes, we next performed GISTIC analysis on 23 potentially actionable genes [15]. We identified six loci with amplifications and nine loci affected by deletions in our actionable gene list in TNBC subtypes that were statistically significant (Supplementary Table S4). Despite being able to identify protein–mutation correlation in only the TP53 gene, these mutations were not accompanied with copy number gain/loss. The PIK3CA mutations in LAR subtype were associated with the copy number amplifications. 

Among other actionable genes, significant copy number amplifications were observed in AKT3 (*q* = 0.001), CCNE1 (*q* = 0.00001) and RPTOR in TNBC subtypes (*q* = 0.04). Significant copy number deletions were identified in the DNA repair gene ATM (*q* = 0.01) (*q* = 0.009), hormone signaling targets PGR (*q* = 0.02), ESR1 (*q* = 0.01) and ERBB3 (*q* = 0.03), tumor suppressor gene RB1 (*q* = 0.0012), and proliferative signaling target CDKN2A (*q* = 0.0005) in TNBC subtypes (Supplementary Table S5). Previously, we identified a significant association of CNAs in 36/49 (73%) genes that were significantly associated with RNA expression in breast cancer subtypes [14]. Here, in our reverse approach analysis, we found a significant association of CNAs with RNA in 8/23 (35%) and proteins in 9/23 (39%) genes, suggesting that correlated copy numbers, mRNA, and protein levels can refine the candidate driver list among TNBC and subtypes (Figure 4b and Supplementary Table S5). Among nine amplifications/deletions, most of these copy number events encompass known oncogenes that showed significant association with protein expression. In mutation analysis, we found the two most frequent mutations, TP53 and PIK3CA, in all TNBC subtypes and only TP53 truncating mutations are significantly correlated with protein expression. These results suggest that CNAs have a more substantial contribution than mutations in TNBC tumors, and that CNAs could thus serve as an oncogenic driver in TNBC. Many genes whose copy number alterations were significantly associated with RNA expression level were also identified at protein levels, such as RPTOR in BL1, EGFR, JAK2, RB1 and CDKN2A in LAR, and MAP2K1 in M subtypes. 

Taken together, these data reveal that structural genomic alterations affect transcription and protein expression, but that this effect is gene specific in different TNBC subtypes.

### 3.5. Molecular Mechanisms Underlying Drug Sensitivity/Resistance 

We identified alterations in multiple signaling pathways, including PI3K/Akt, MAPK, NOTCH, JAK, EGFR, and cell cycle pathways revealing that TNBC chemoresistance is based on multilayered complicated machinery. Based on the results of single molecular/omic level data, we dissect the mechanism of drug sensitivity and resistance of these molecular signatures. We explored the pharmacogenomic data from GDSC in 3 BL1, 1 BL2, 2 LAR, and 1 M TNBC cell lines. We curated the drug sensitivity/resistance results of the markers identified in our results with mRNA–CNA, protein–CNA, and protein–mRNA–CNA associations and found 16 anticancer drug candidates associated with drug sensitivity/resistance. The EGFR is one of the common targets identified in all three associations (mRNA–CNA, protein–CNA, and protein–mRNA–CNA) and is strongly associated with sensitivity to Food and Drug Administration (FDA)-approved EGFR inhibitor lapatinib and other drugs in the BL1, BL2, and LAR cell lines. Another important identified target in our data is AKT1, where mRNA expression is associated with CNA and the potential mechanism of drug sensitivity is AKT inhibitors sensitive to two common drugs, afuresertib and ipatasertib in the BL1 and LAR cell lines, respectively. Interestingly, the M cell line (CAL-120) displays high levels of drug resistance for all molecular signatures. Although our integrated proteogenomic approach (multi-omic analysis) identifies some potential drug sensitive/resistance mechanisms, these results are based on a small number of TNBC cell lines that could not fully capture TNBC heterogeneity (Supplementary Figure S3a). Overall, these results demonstrate that TNBC subtypes are markedly heterogeneous at the single omics level, and it is still a challenge to identify the specific molecular mechanism in TNBC. 

To further validate the markers identified in our integrated proteogenomics analysis, we validated these results using mass spectrometry CPTAC-related mutations, RNA expression, CNAs, and protein data in 29 basal patients. We observed a similar trend of associations between the protein, RNA, mutation, and CNA levels. For instance, at protein–mutation correlation, only TP53 truncating mutations were significantly associated with protein expression. We observed the change at the protein level associated with CNAs in PIK3CA, RPTOR, and RB1 genes. Likewise, for protein–RNA–CNA correlation, a significant association was observed for PIK3CA and RPTOR genes (Supplementary Figure S3b).

## 4. Discussion

We carried out an in-silico analysis using genomic, transcriptomic, and proteomic TCGA data in four molecular subtypes of TNBC defined by Lehmann et al. [6]. To determine if the genomic alterations can be directly inferred from proteomic data, we characterized the expression levels and post-translational modification of key cancer-related proteins, which are important candidates for drug targets. We explored the linkage of the proteomics data with high throughput genomics (DNA mutations and CNAs) and transcriptomics data to uncover the process that occurs at the protein level and how the proteomics data can add to our understanding of driving mechanisms in four TNBC subtypes. Our work adds to the list of potentially predictive alterations in TNBC to move us closer towards improved precision medicine for this devastating disease.

Our proteogenomics approach is based on the following three steps: (a) to depict the protein and phosphoprotein levels of 23 potentially actionable genes in four TNBC subtypes, (b) to elucidate the effects of genomic alterations (DNA mutations and CNAs) on protein level or to identify the driver gene mutations/CNAs associated with abnormal protein expression, and (c) to evaluate whether only genomics or transcriptomics data can provide a proteomic view of TNBC subtypes. We identified distinct copy number signatures among 23 genes across TNBC subtypes. Both high-level amplifications and deletions were associated with BL1 and BL2 subtypes. LAR subtype was only associated with amplifications and the M subtype with deletions, suggesting that different mechanisms underlie chromosome instability across the spectrum of TNBC. Taking a proteomics-based approach, the key finding in this study is that the correlation of proteomics data with copy number and mutations prioritize CNAs as the genomic drivers over mutations and therefore could be potential therapeutic targets in TNBC subtypes. We, and others, have previously reported that mRNA expression is associated with CNAs in breast tumors [14,29]. However, the mRNA–CNA association will have a relative abundance of transcripts and many of these are not or partially expressed to proteins [30]. In all four TNBC subtypes, we identified a high correlation between protein–mRNA in 22/23 (96%) genes, protein–CNA correlation in 9/23 (39%) targets, mRNA–CNA correlation in 10/23 (43%), and protein–mRNA–CNA in 4/23 (17%) genes. These data suggest that there is a stronger bonding between protein–CNA than protein–mRNA–CNA. These results suggest that a number of genomic alterations are translated to the protein level and that CNAs are attenuated by post-transcriptional regulation. The remaining changes in protein levels could be reflected by other mechanisms, such as the concerted action of epigenetic variations, including chromatin regulation, histone post-translation modifications and DNA methylation. Surprisingly, we found a correlation between proteins and mutations in only 1/23 (4%) genes and no significant correlation for protein–RNA–mutation, indicating that not all mutations in potentially actionable genes are expressed.

The major goal of this study was the identification of specific aberrations from the proteome, a site of most therapeutic interventions, by connecting proteomics to genomics data. Amongst these TNBC subtypes, we found some differences in LAR tumors in comparison with other TNBC subtypes. LAR tumors were associated with a higher mutational burden in the PIK3CA gene, with significant mRNA overexpression. However, the correlation was more variable at the protein level. Strikingly, we identified a relatively weak proteogenomic link for the PIK3CA gene between protein–RNA, protein–mutation, and CNA–protein, suggesting that genomic alterations (mutations/CNAs) in the PIK3CA gene are not or are only partially translated to the protein level. In addition, we identified a significant association of protein expression with copy number gain and high-level amplifications for EGFR, RB1, CDKN2A, and JAK2 genes in the LAR subtype. The proteogenomic link with RNA expression and copy number amplification/loss was particularly significant for the other actionable genes, including PIK3CA for BL1, RPTOR and CDKN2A for BL2, and MAP2K1 for M subtypes. These genes are predicted to be the targets of drug inhibitors, including CDKs, PI3K, TKI, and mTOR. We also integrated the proteomics and genomics data with drug sensitivity/resistance profiles from TNBC cell lines to identify potential molecular mechanisms responsible for variations in drug response. The identification of molecular mechanisms is based on cell-line data and does not guarantee a similar drug response in TNBC patients. Additionally, though the data suggest that our reverse analysis workflow is a reasonable approach for uncovering genomic alterations that are susceptible to anticancer drugs, this scheme needs to be validated in a clinical setting.

The CPTAC has produced mass spectrometry-based proteomics data from TCGA tumors for breast, colon, and ovarian cancer and demonstrated that the proteogenomic linkage uncovers the potential oncogenic drivers, rather than genomic analysis alone [13,31,32]. However, leveraging the genomic information by dissecting the cancer proteome to the genome to prioritize the genetic entities that drive TNBC to an aggressive phenotype has yet to be explored. Although it is beyond the scope of our perspective, the remarkable response of immunotherapy has changed the treatment paradigm of several malignancies, including TNBC, but only a minority of patients respond, thus highlighting the need for novel therapeutic strategies. The TCGA has generated a comprehensive catalog that provides remarkable insights into the breast cancer genome, transcriptome, and proteome. We have also validated this strategy in a basal cohort of the CPTAC dataset. The result from this study shows that our framework, which prioritizes potential therapeutic targets by working backward from proteins to RNA and then to DNA, provides a more precise picture of the TNBC proteogenomic landscape by narrowing the gap between cancer genotype and phenotype. This strategy could also help in the identification of putative neoantigens and effective personalized cancer treatment. However, there are some limitations to our approach. The RPPA platform used by the TCGA has protein expression levels for ~200 antibodies targeting ~150 genes. From our 49 actionable gene list, RPPA data were available from only 23 genes for proteogenomic analysis, partly accounting for very few numbers of genes with protein–RNA–DNA/CNAs correlation, and that this also accounts for the small number of cases (29–33) analyzed in each TNBC subtype. Moreover, there are post-transcriptional and post-translation mechanisms, such as microRNA regulation, acetylation, and methylation that also reflect active cellular functions and which have not been addressed in this analysis. While RPPA provides a high throughput strategy by which to detect proteomic biomarkers in large numbers of specimens, the low correlation between gene level data for RNA and DNA is noteworthy. The lower limits of detection of protein and antibody specificity for variant proteins may be confounders. 

## 5. Conclusions

Our integrated proteogenomics data provide valuable insights into how the genomic alterations can be inferred only from proteomics using a reverse analysis approach or how CNAs influence the proteome. Additionally, our data also yielded several insights into TNBC biology at the mutation, copy number, RNA expression, protein expression, pathway levels, and drug sensitivity/resistance molecular mechanisms. Our proteogenomic approach helps to identify the oncogenic drivers, such as copy number aberrations in TNBC. However, more caution should be exercised when detecting cancer drivers based on post-transcriptional mechanisms. Though we found that most of our actionable proteins were linked to RNA expression, changes in the transcription levels are poor predictors of protein activity changes. In the clinic, there is increased attention towards genomic profiling of tumor biopsy and liquid biopsy samples. Our study in TNBC shows that only rarely does a DNA mutation correlate with protein expression. This multi-omics approach, if appropriately implemented in the clinical setting, could effectively fill the critical gaps that occur with the single-omics approach. 

## Figures and Tables

**Table 1:** Characteristics of TNBC patients included in Lehmann’s classification.

	BL1 (n = 33), n (%)	BL2 (n = 22), n (%)	LAR (n = 29), n (%)	M (n = 29), n (%)	*p*-Value
**Age**					0.92
**Mean + SD**	11.0 + 5.1	7.3 + 4.3	9.6 + 3.4	9.4 + 2.6	
26–40	4 (12.1%)	0 (0.0%)	4 (13.8%)	5 (17.2%)	
41–60	21 (63.6%)	15 (68.2%)	16 (55.2%)	14 (48.3%)	
61–90	8 (24.2%)	7 (31.8%)	9 (31%)	10 (34.5%)	
**Ethnicity**					0.98
Hispanic or Latino	0 (0.0%)	3 (13.6%)	1 (3.4%)	1 (3.4%)	
Not Hispanic or Latino	29 (87.9%)	19 (86.4%)	25 (86.2%)	25 (86.2%)	
NA	3 (9.1%)	0 (0.0%)	3 (10.3%)	3 (10.3%)	
NE	1 (3.0%)	0 (0.0%)	0 (0.0%)	0 (0.0%)	
**Race**					0.97
White	19 (57.6%)	15 (68.2%)	21 (72.4%)	19 (65.5%)	
Black or African American	8 (24.2%)	1 (4.5%)	5 (17.2%)	8 (27.6%)	
American Indian or Alsaka Native	0 (0.0%)	0 (0.0%)	0 (0.0%)	0 (0.0%)	
Asian	4 (12.1%)	6 (27.3%)	3 (10.3%)	2 (6.9%)	
NA	1 (3.0%)	0 (0.0%)	0 (0.0%)	0 (0.0%)	
NE	1 (3.0%)	0 (0.0%)	0 (0.0%)	0 (0.0%)	
Tumor Stage					0.99
Early (Stage I–III)	32 (97.0%)	21 (95.5%)	28 (96.6%)	28 (96.6%)	
Metastatic	0 (0.0%)	0 (0.0%)	0 (0.0%)	1 (3.4%)	
NA	1 (3.0%)	1 (4.5%)	1 (3.4%)	0 (0.0%)	
**Neoadjuvant treatment**					0.99
Yes	0 (0.0%)	0 (0.0%)	0 (0.0%)	0 (0.0%)	
No	33 (100%)	22 (100%)	29 (100%)	29 (100%)	
NA	0 (0.0%)	0 (0.0%)	0 (0.0%)	0 (0.0%)	
**TNM Stage**					
*T Stage*					0.97
T1	4 (12.1%)	4 (18.2%)	7 (24.1%)	6 (20.7%)	
T2	26 (78.8%)	14 (63.6%)	18 (62.1%)	18 (62.1%)	
T3	2 (6.1%)	2 (9.1%)	2 (6.9%)	5 (17.2%)	
T4	1 (3.0%)	2 (9.1%)	2 (6.9%)	0 (0.0%)	
TX	0 (0.0%)	0 (0.0%)	0 (0.0%)	0 (0.0%)	
*N Stage*					0.99
N0	23 (69.7%)	15 (68.2%)	14 (48.3%)	20 (69.0%)	
N1	9 (27.3%)	3 (13.6%)	8 (27.6%)	6 (20.7%)	
N2	0 (0.0%)	3 (13.6%)	4 (13.8%)	2 (6.9%)	
N3	1 (3.0%)	1 (4.5%)	3 (10.3%)	1 (3.4%)	
NX	0 (0.0%)	0 (0.0%)	0 (0.0%)	0 (0.0%)	
*M Stage*					0.96
M0	31 (93.9%)	20 (90.9%)	26 (89.7%)	25 (86.2%)	
M1	0 (0.0%)	0 (0.0%)	0 (0.0%)	2 (6.9%)	
MX	2 (6.1%)	2 (9.1%)	3 (10.3%)	2 (6.9%)	
**Hormone Status**					
*ER status*					0.99
ER-positive	0 (0.0%)	2 (9.1%)	0 (0.0%)	2 (6.9%)	
ER-negative	31 (93.9%)	20 (90.9%)	28 (96.6%)	26 (89.7%)	
NE	2 (6.1%)	0 (0.0%)	1 (3.4%)	1 (3.4%)	
Indeterminate	0 (0.0%)	0 (0.0%)	0 (0.0%)	0 (0.0%)	
Equivocal	0 (0.0%)	0 (0.0%)	0 (0.0%)	0 (0.0%)	
*PR status*					0.99
PR-positive	0 (0.0%)	0 (0.0%)	0 (0.0%)	0 (0.0%)	
PR-negative	31 (93.9%)	22 (100%)	28 (96.6%)	27 (93.1%)	
NE	2 (6.1%)	0 (0.0%)	1 (3.4%)	2 (6.9%)	
Indeterminate	0 (0.0%)	0 (0.0%)	0 (0.0%)	0 (0.0%)	
Equivocal	0 (0.0%)	0 (0.0%)	0 (0.0%)	0 (0.0%)	
*HER2 status*					0.96
HER2-positive	2 (6.1%)	0 (0.0%)	3 (10.3%)	3 (10.3%)	
HER2-negative	19 (57.6%)	16 (72.7%)	19 (65.5%)	15 (51.7%)	
NE	6 (18.2%)	1 (4.5%)	3 (10.3%)	5 (17.2%)	
Indeterminate	0 (0.0%)	1 (4.5%)	0 (0.0%)	0 (0.0%)	
Equivocal	6 (18.2%)	4 (18.2%)	4 (13.8%)	5 (17.2%)	
NA	0 (0.0%)	0 (0.0%)	0 (0.0%)	1 (3.4%)	

## Data Availability

The data analyzed in the current study are available in TCGA GDC data portal, Broad Institute GDAC Firehose, and cBioportal platform.

## Supplementary Materials

The following supporting information can be downloaded at https://www.mdpi.com/article/10.3390/cancers16030516/s1, Figure S1: Protein and phosphoprotein expression of potentially actionable genes; Figure S2: The most altered actionable genes in TNBC subtypes and their effect on the protein; Figure S3: In silico analysis of pharmacogenomic interactions in TNBC and comparison of RPPA TCGA versus mass spectrometry CPTAC data; Table S1: Positive and negative protein–protein correlation in TNBC subtypes; Table S2: OncodriveFML analysis for the detection of candidate driver genes in TNBC subtypes; Table S3: Positive protein-RNA correlation in TNBC subtypes; Table S4: Significant amplified and deleted regions in TNBC subtypes, identified by GISTIC which contains potentially actionable genes from our list; Table S5: Significant association of CNAs with protein expression in actionable genes in TNBC subtypes.
