# Supplementary material for: Integrated Proteogenomic Analysis Reveals Distinct Potentially Actionable Therapeutic Vulnerabilities in Triple-Negative Breast Cancer Subtypes
Source: Cancers (Basel). 2024 Jan 25;16(3):516. doi: 10.3390/cancers16030516 (PMC10854633; doi:10.3390/cancers16030516)
Supplement: Supplementary file 1 [file cancers-16-00516-s001.zip › Supplementary figure legends.pdf]

Integrated Proteogenomic Analysis Reveals Distinct Potentially Actionable Therapeutic Vulnerabilities in Triple-Negative Breast Cancer Subtypes

Pushpinder Kaur [1,2], Alexander Ring [3], Tania B. Porras [4], Guang Zhou [5], Janice Lu [2,6], Irene Kang [2,6] and Julie E. Lang [1,2,5,*]

[1] Department of Surgery, Keck School of Medicine, University of Southern California, Los Angeles, CA 90033, USA
[2] Norris Comprehensive Cancer Center, University of Southern California, Los Angeles, CA 90033, USA
[3] Department of Medical Oncology and Hematology, University Hospital Zürich, 8091 Zurich, Switzerland
[4] Cancer and Blood Disease Institute, Children Hospital Los Angeles, University of Southern California, Los Angeles, CA 90027, USA
[5] Division of Breast Services, Department of General Surgery, Digestive Disease and Surgery Institute, Department of Cancer Biology, Lerner Research Institute, Cleveland Clinic, Cleveland, OH 44195, USA
[6] Division of Medical Oncology, Department of Medicine, University of Southern California Norris Cancer Center, University of Southern California, Los Angeles, CA 90033, USA
* Correspondence: langj2@ccf.org; Tel.: +1-216-636-2843

Supplementary figure legends:

Supplementary Figure S1: Protein and phosphoprotein expression of potentially actionable genes. (a) The bar graph shows no significant variation in the protein expression of individual genes. The 2-way ANOVA test was used for average protein expression for individual and all 23 proteins together and individual genes in each TNBC subtype. **(b)** The bar graph shows no significant variation in the phosphoprotein levels of individual genes. The 2-way ANOVA test was used for average phosphoprotein levels for individuals and all 11 phosphoproteins together in each TNBC subtype.

Supplementary Figure S2: The most altered actionable genes in TNBC subtypes and their effect on the protein. (a) Quantile-quantile (QQ) plots to show the top-ranking significant

actionable genes using the set of somatic mutations for each TNBC subtype. The x- and y-axis showed the expected and observed distribution of FM bias p values of actionable genes. Blue dots- genes with at least one somatic mutation. Red dotted line- coincident values of expected and observed distribution of FM bias p values of actionable genes. Genes identified as significant with a q-value of <0.1 are named in red and a q-value <0.25 in green color. **(b)** Boxplots showing the protein expression level of TP53 in wild type (WT) (grey), missense (MS) (red), truncating mutation (TM) (blue), splice site (green), and frameshift (FS) (black) mutations in cases across TNBC. The y-axis showed the protein expression level of wild-type and mutated cases.

Supplementary Figure S3: ***In silico*** **analysis of pharmacogenomic interactions in TNBC and comparison of RPPA TCGA versus mass spectrometry CPTAC data.** **(a)** Summary of pharmacologic interactions identified in TNBC cell lines for the proteogenomic markers identified in our reverse analysis approach. **(b)** Venn diagram of common and distinct markers in the TCGA and CPTAC datasets showing associations of molecular features (mRNA-CNA, protein-CNA, and protein-mRNA-CNA).
