# Supplementary material for: Integrated Proteogenomic Analysis Reveals Distinct Potentially Actionable Therapeutic Vulnerabilities in Triple-Negative Breast Cancer Subtypes
Source: Cancers (Basel). 2024 Jan 25;16(3):516. doi: 10.3390/cancers16030516 (PMC10854633; doi:10.3390/cancers16030516)

Supplementary fig. S1

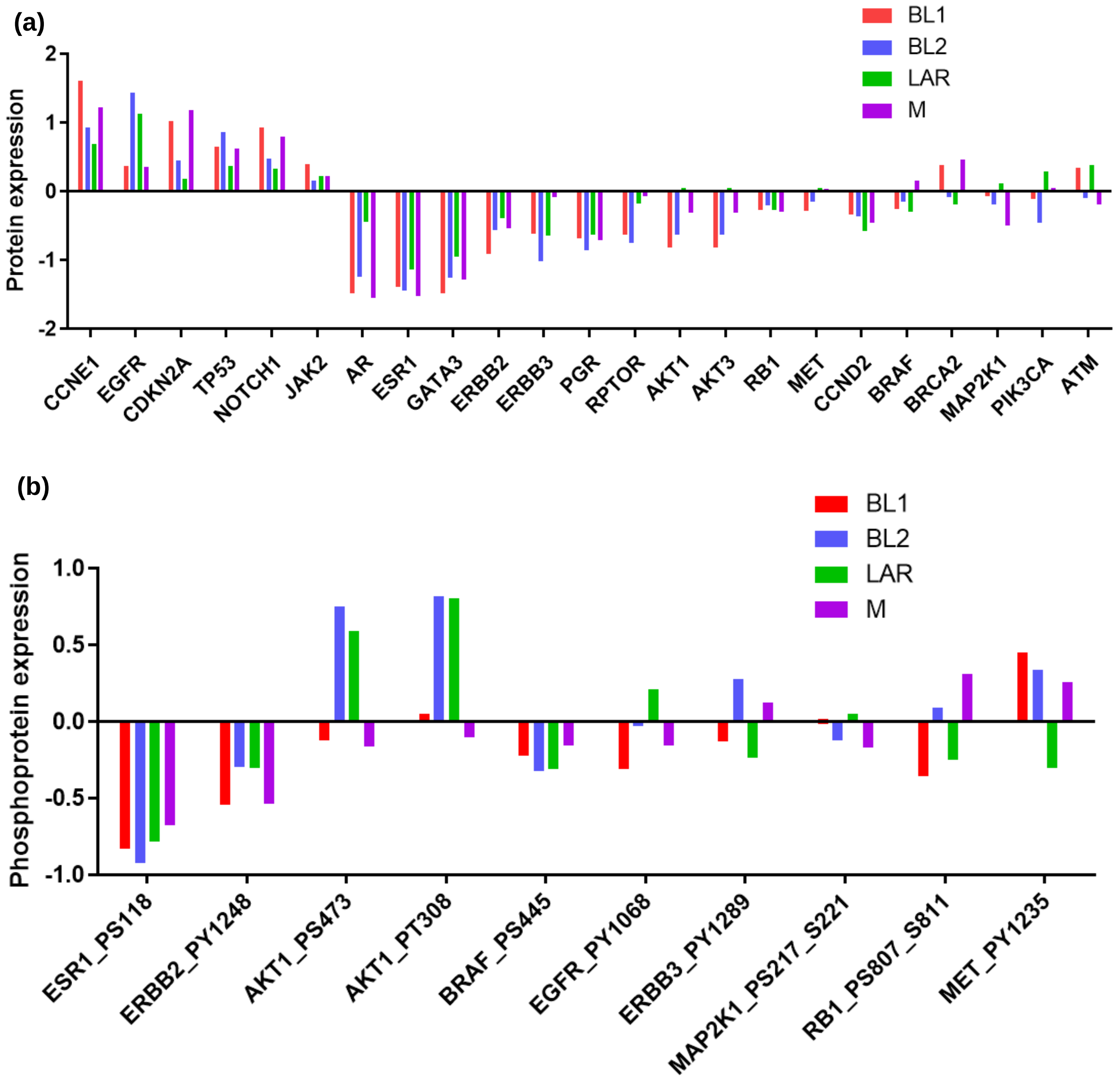

Supplementary fig. S2

(a)

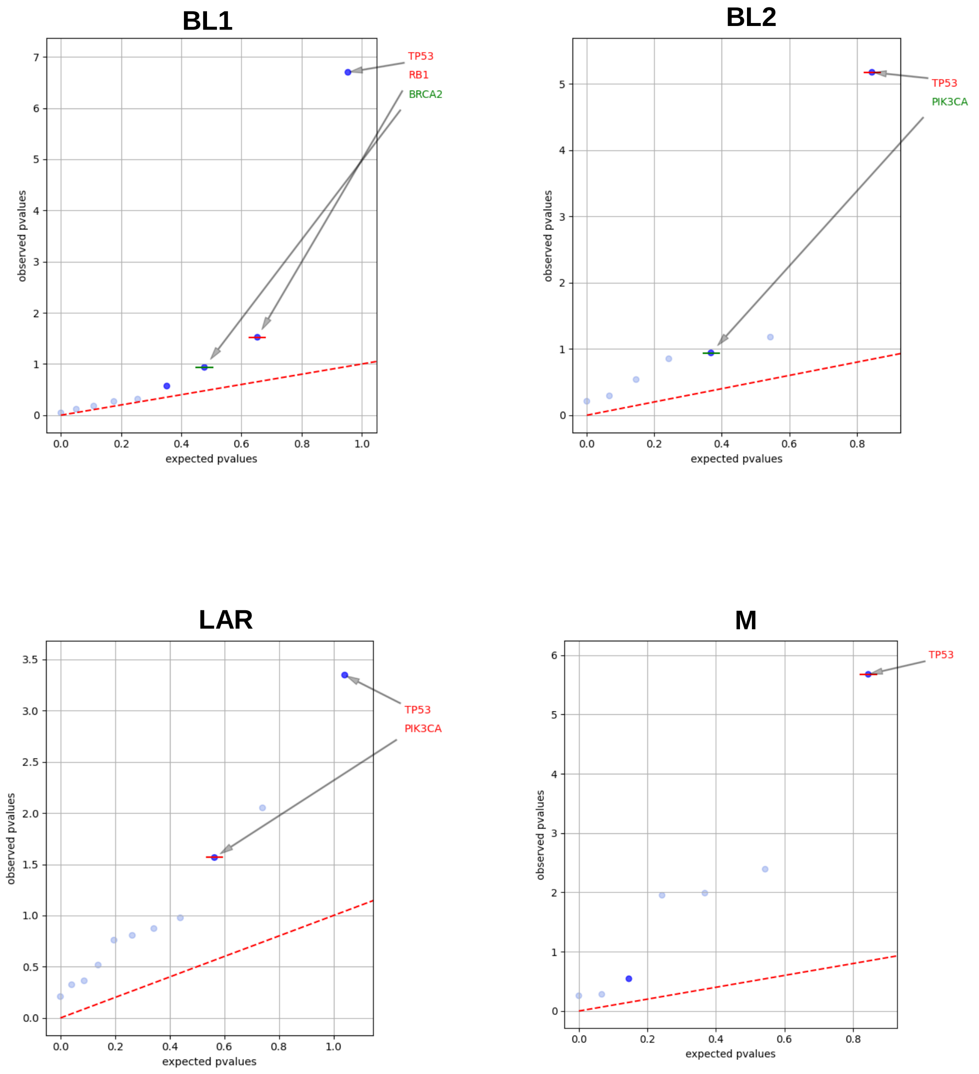

(b)

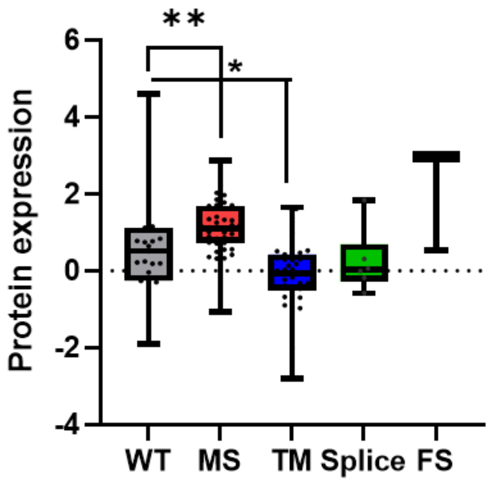

Supplementary fig. S3

(a)

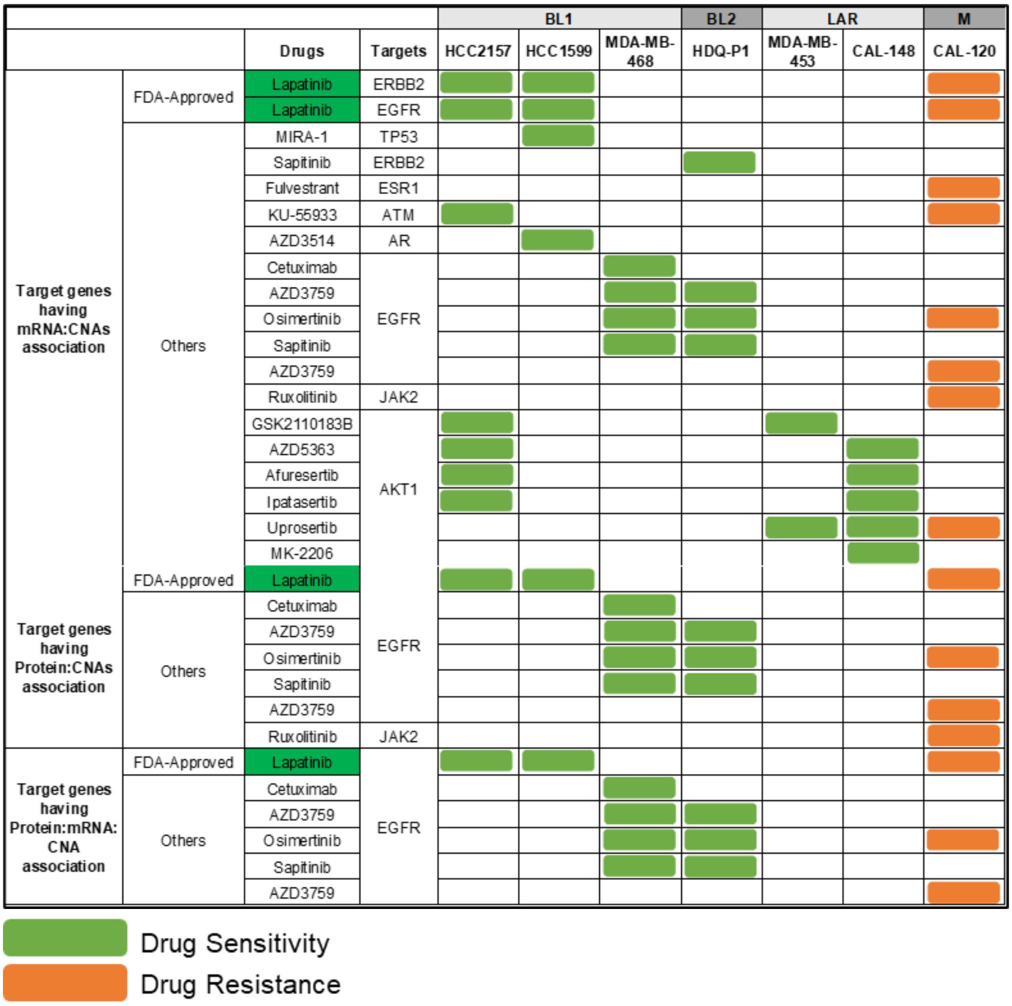

(b)

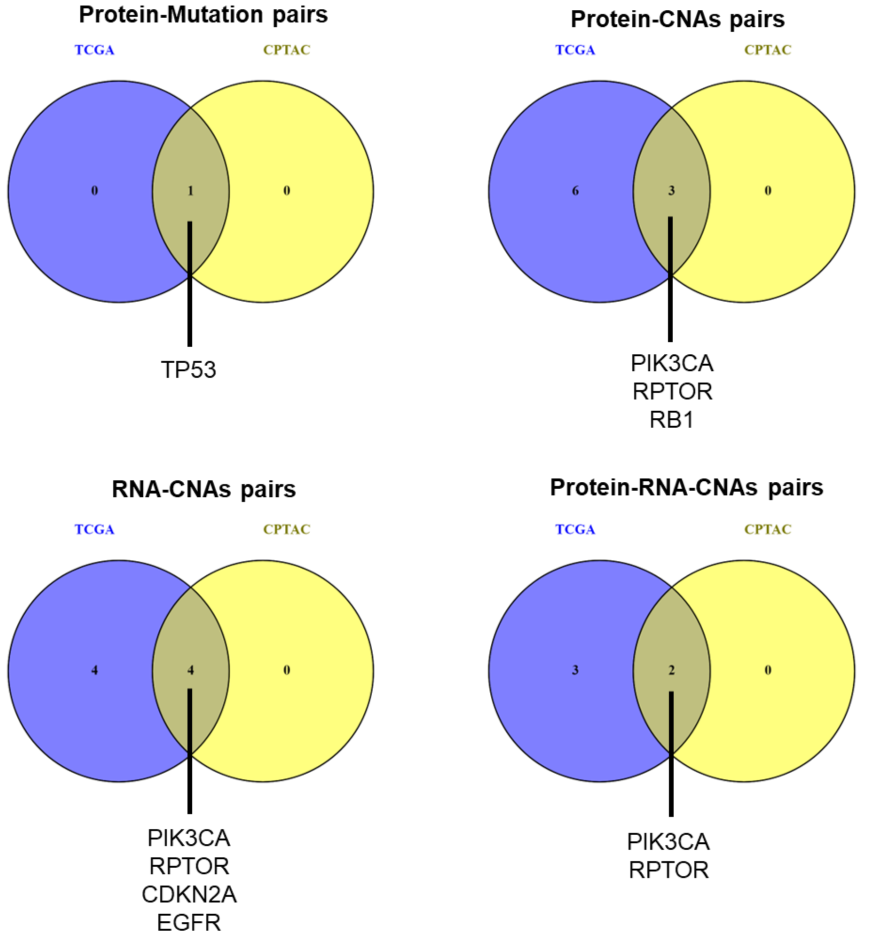

Supplement: Supplementary file 1 [file cancers-16-00516-s001.zip › Supplementary figures.pdf]
